# New Insights on Signal Propagation by Sensory Rhodopsin II/Transducer Complex

**DOI:** 10.1038/srep41811

**Published:** 2017-02-06

**Authors:** A. Ishchenko, E. Round, V. Borshchevskiy, S. Grudinin, I. Gushchin, J. P. Klare, A. Remeeva, V. Polovinkin, P. Utrobin, T. Balandin, M. Engelhard, G. Büldt, V. Gordeliy

**Affiliations:** 1 Institute of Complex Systems (ICS), ICS-6: Structural Biochemistry, Research Centre Jülich, 52425 Jülich, Germany; 2 Institute of Crystallography, University of Aachen (RWTH), Jägerstraße 17-19, 52056 Aachen, Germany; 3 Institut de Biologie Structurale J.-P. Ebel, Université Grenoble Alpes-CEA-CNRS, F-38000 Grenoble, France; 4 CNRS, Laboratoire Jean Kuntzmann, BP 53, Grenoble Cedex 9, France; 5 Moscow Institute of Physics and Technology, 141700 Dolgoprudniy, Russia; 6 NANO-D, INRIA Grenoble-Rhone-Alpes Research Center, 38334 Saint Ismier Cedex, Montbonnot, France; 7 Max-Planck Institute of Molecular Physiology, 44227 Dortmund, Germany; 8 Department of Physics, University of Osnabrück, Barbarastrasse 7, D-49069 Osnabrück, Germany

## Abstract

The complex of two membrane proteins, sensory rhodopsin II (NpSRII) with its cognate transducer (NpHtrII), mediates negative phototaxis in halobacteria *N. pharaonis*. Upon light activation NpSRII triggers a signal transduction chain homologous to the two-component system in eubacterial chemotaxis. Here we report on crystal structures of the ground and active M-state of the complex in the space group I2_1_2_1_2_1_. We demonstrate that the relative orientation of symmetrical parts of the dimer is parallel (“U”-shaped) contrary to the gusset-like (“V”-shaped) form of the previously reported structures of the NpSRII/NpHtrII complex in the space group P2_1_2_1_2, although the structures of the monomers taken individually are nearly the same. Computer modeling of the HAMP domain in the obtained “V”- and “U”-shaped structures revealed that only the “U”-shaped conformation allows for tight interactions of the receptor with the HAMP domain. This is in line with existing data and supports biological relevance of the “U” shape in the ground state. We suggest that the “V”-shaped structure may correspond to the active state of the complex and transition from the “U” to the “V”-shape of the receptor-transducer complex can be involved in signal transduction from the receptor to the signaling domain of NpHtrII.

Archaebacterial photoreceptors mediate phototaxis by regulating cell motility through two-component signaling cascades like those found in chemotaxis signaling chains of enteric bacteria^1^. The photoreceptor sensory rhodopsin II from *N. pharaonis* (NpSRII) in complex with its cognate transducer NpHtrII serves as a model system for studying transmembrane signal transfer. This complex displays a 2:2 stoichiometry where two transducers are flanked by two receptors. The long rod-shaped cytoplasmic domain consists of two HAMP domains (present in Histidine kinases, Adenyl cyclases, Methyl-accepting proteins and Phosphatases^2^,^3^), which are followed by a methylation domain^4^ involved in the adaptation processes and a signaling domain which harbors the CheW/CheA binding site^5^. HAMP subunits contain two amphiphilic helices (AS1 and AS2) joined by a non-helical connector. The first HAMP domain (HAMP1, amino acid residues 83–135) and the second HAMP domain (HAMP2, amino acid residues 157–210) are connected by an α-helical linker (Fig. 1). Despite high abundance of HAMP domains in different classes of proteins, their role and function is still unclear^2^,^6^,^7^. For understanding the signal transfer from the receptor to the transducer it is mandatory to determine the exact structure of the binding surface connecting NpSRII with its cognate transducer. Currently, the structure of the truncated membrane domain has been determined, however for the adjacent proximal region only indirect experimental evidences^8^,^9^,^10^,^11^ are available. Here, we report on new crystal structures of the ground and active M-state of NpSRII/NpHtrII_1–157_ complexes, in the space group I2_1_2_1_2_1_. Interestingly, the orientation of symmetrical parts of the dimer of two proteins is “U”-shaped contrary to the “V”-shaped quaternary structure reported previously (space group P2_1_2_1_2)^12^. The detailed structure of the retinal-binding pocket remains the same. Our computer modeling data reveals that the HAMP domain in its resting conformation^6^ acquires complimentary intermolecular interactions with the receptor only in the “U”-shaped conformation. We suggest a biological relevance of this finding and propose that activation of the receptor/transducer complex involves a transition from the “U”- to “V”-shaped quaternary structures.

The previously published X-Ray diffraction data^12^,^13^ on the NpSRII/NpHtrII complex only showed electron densities for the transmembrane domain of the transducer but not for its adjacent HAMP domain. These crystals of the wild type NpSRII in complex with the shortened NpHtrII (NpHtrII_1–157_ and NpHtrII_1–114_) belonging to the space group P2_1_2_1_2 revealed an overall “V”-shaped topology with an opening to the cytosplasmic side. Our homology modeling of the HAMP domain attached to NpHtrII of the “V”-shaped structure did not show any direct contacts that would explain the lack of an electron density in this region. Contrary to these findings, the experiments by various biophysical methods^8^,^9^,^10^,^11^ demonstrated an intimate interaction of TM2 of the transducer with the E-F loop of the receptor. This discrepancy could be accounted for by the influence of crystallization conditions on the global arrangement of proteins. Previous studies have already shown that modification of crystallization conditions can result in different functional conformations of a protein^14^.

## Results

In an intensive crystallization screening we obtained two new crystal forms displaying space groups with the I2_1_2_1_2_1_ and P6_4_ symmetry. Resolution for the both ground and M states was 1.9 Å in the case of the I2_1_2_1_2_1_ space group and 2.5 Å in the case of the P6_4_ space group. Since no substantial discrepancies were found between ground state protein structures in both space groups, we will discuss the structure in the I2_1_2_1_2_1_ space group as it has higher resolution. When comparing wild type structures solved in the I2_1_2_1_2_1_ and previously solved in the P2_1_2_1_2 space groups it is seen that the structure of NpSRII is nearly the same (with backbone RMS deviation of 0.22 Å for ordered regions) and is highly similar to the structure of NpSRII when crystallized alone (with backbone RMS deviation of 0.27 Å for ordered regions)^15^,^16^,^17^.

### Signal transduction mechanism

The generally accepted mechanism^5^,^18^,^19^,^20^,^21^ of the signal transduction in the complex is the following. After isomerization, the retinal looses a proton from the Schiff base, which is then translocated to the Schiff base counterion^22^ (Asp75 in case of NpSRII). This event leads to the charge rearrangement in the active site of the molecule, disorder of some water molecules of the active site and, therefore, breaking of the hydrogen bonds. This hydrogen bond network integrates helices of resting state NpSRII into a tight bundle and diminishing the strength of this H-bond system results into displacements of the transmembrane helices, in particular F and G, both parallel and perpendicular to the membrane. These shifts change the hydrophobic area of the receptor and have important implications for the signal transduction, as it will be discussed below.

**Figure 2:**
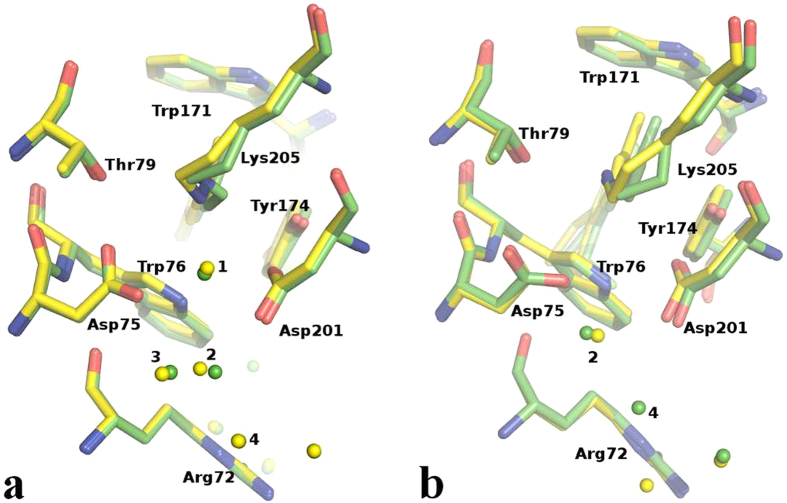
Structures of NpSRII active site. (**a**) Structural differences between NpSRII ground state in I2_1_2_1_2_1_ space group (yellow) and in P2_1_2_1_2 space group (green) and (**b**) between NpSRII M state in I2_1_2_1_2_1_ space group (yellow) and in P2_1_2_1_2 space group (green).

**Figure 3:**
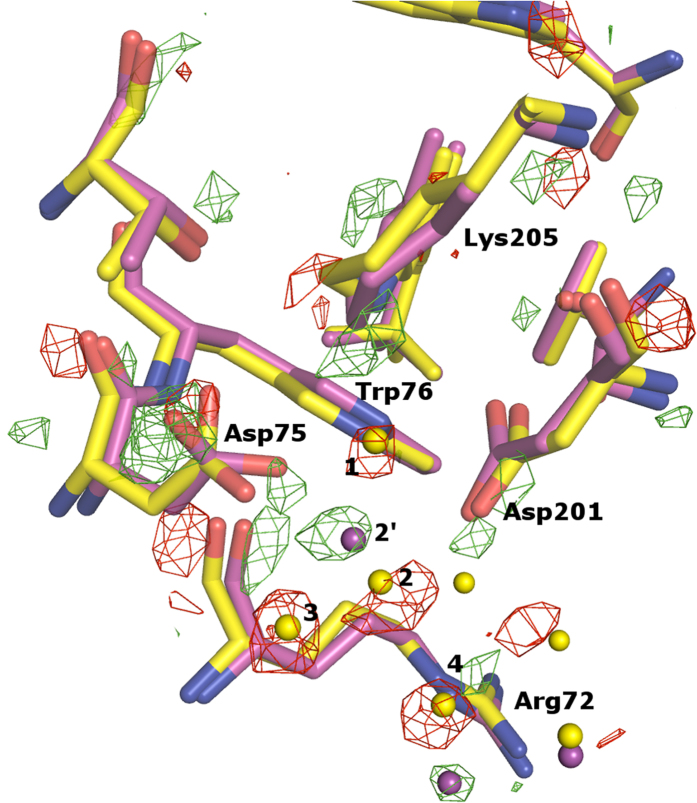
Structural differences between NpSRII ground state (yellow) and the M state intermediate (magenta) in the vicinity of the retinal Schiff base including water molecules, Lys205, Asp75, Asp201, Arg72, Thr79, Trp76, Trp171. Water molecules are depicted as spheres. Electron density maps are contoured at −3σ and 3σ.

Having two data sets from different space groups (I2_1_2_1_2_1_ and P2_1_2_1_2), we checked whether different crystal contacts have a structural influence on the retinal-binding site. As can be seen from Fig. 2 there are almost no differences found for the ground state structures (Fig. 2a) as well as for the activated M intermediate (Fig. 2b), indicating that the different crystallization conditions and space groups have little, if any at all, influence on the functionally important regions within NpSRII. This is also substantiated if one analyzes the transition between the ground state and the M state (Fig. 3).

Upon photon absorption by the complex in the space groups I2_1_2_1_2_1_ as in P2_1_2_1_2, the retinal chromophore switches from *all-trans* conformation to 13*-cis* and Lys-Cε simultaneously shifts towards the central water cluster^17^. The side chain of Asp75 is rotated by about 90° from its ground state position, thereby losing the hydrogen bond to Thr79^13^,^23^. Consequently, W3 becomes disordered and disappears in the M state. The pentagon of hydrogen bonds (Asp201-Oδ···W2···W3···Arg72-Nε···W1) does not exist any longer in the M state because molecules W1 and W3 have vanished^23^. Consequently, helices C and G of NpSRII have more freedom to move independently. The water molecule W2′ is located close to Asp75 and Arg72. Thus, the signal is generated by the retinal isomerization and then propagates to the interface between the receptor and the transducer. The signal transmission is driven by the rearrangement of the hydrogen bond network, mediated by water molecules, which leads to the movement of the receptor helices described above due to the loss of connectivity between C and G helices.

The movement of the helix G of NpSRII initiates the movement of TM2 in NpHtrII. All hydrogen bonds, between the receptor and the transducer, observed for the ground state are still intact in the M state. A piston like movement of the cytoplasmic end of TM2 of about 0.5 Å and rotation of 19° (15° in P2_1_2_1_2) are observed and accompanied by alteration in the direction of Tyr199-Asn74 hydrogen bond. The rotation of the transducer’s TM2 has also been observed by EPR spectroscopy^5^. The similarity of the differences between the ground and the M states in the new type of crystals shows an overall robustness of the signal transduction mechanism in the membrane part of the complex.

### Quaternary structure of the complex

**Figure 4:**
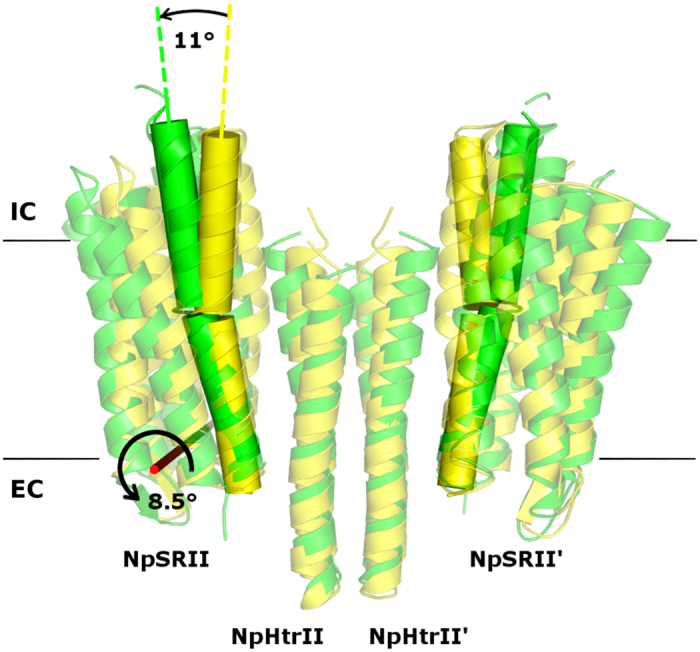
“V”- and “U”-shapes of the wild type dimer as seen along the membrane. Helices G are shown as cylinders. The angle between the two helices G belonging to the same NpSRII molecule in the “U”- and “V” shapes is shown. The axis of rotation of NpSRII as a rigid body during the transition from the “U”- to “V”-shape is shown in red and the corresponding angle is presented. The structure solved in the I212121 space group (“U”-shape, PDB ID 4GY6) is shown in yellow, the structure solved in the P21212 space group (“V”-shape, PDB ID 1H2S) is shown in green. Primes denote symmetry mates. Solid black lines illustrate the approximate membrane boundaries with “IC” designating the intracellular side and “EC” designate the extracellular side.

**Figure 5:**
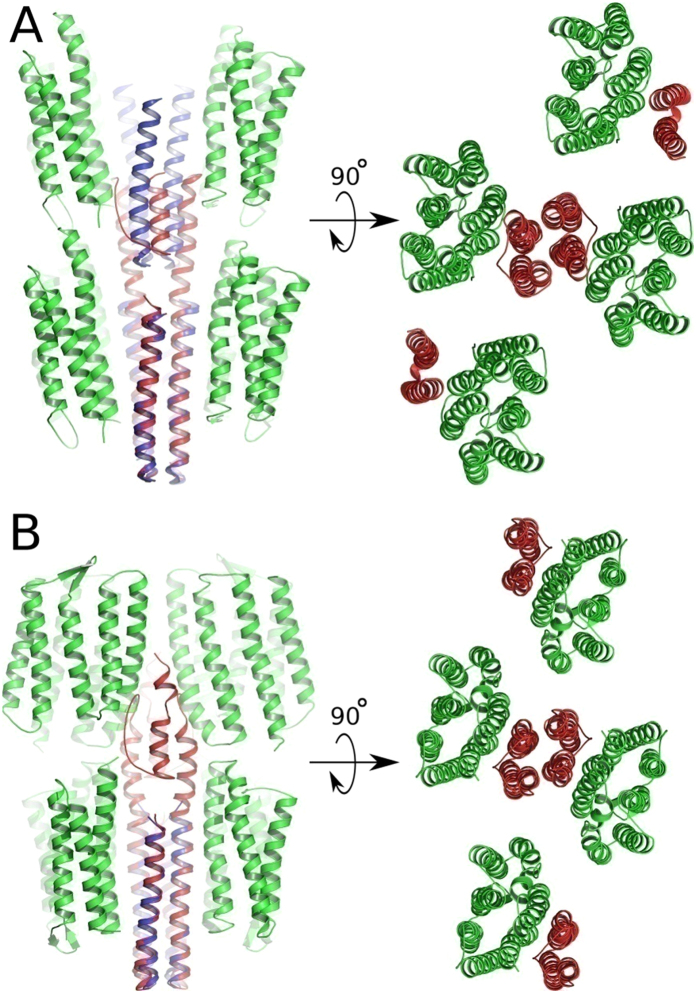
(**A**) “V”-shaped NpSRII/NpHtrII complex in P21212 space group. (**B**) “U”-shaped NpSRII/NpHtrII complex in I212121 space group. Figures on the left show two subsequent crystal layers as seen along the membrane plane. Figures on the right show a single crystal layer as seen perpendicular to the membrane plane. NpSRII molecules are shown in green; parts of NpHtrII molecules resolved in the crystal structure are shown in blue. Models of NpHtrII that continue to the HAMP domain are shown in red. Residues 83–136 in these models are modeled by homology with NMR structure 2ASW. In (**A**) a big steric clash can be seen between the model of the HAMP domain from one crystal layer and the corresponding NpHtrII molecule from the other crystal layer. There are no observed steric clashes between the model of the HAMP domain from one layer and NpSRII/NpHtrII molecules from the other layer in (**B**).

Having established that the fine structure of the proteins itself in the complex in the two crystal forms is unperturbed, we analyzed the overall structures in the crystal lattices. It is evident from Fig. 4 that the two receptors are oriented nearly parallel to the transducer and form a “U”-shaped binding crevice in the new crystal form I2_1_2_1_2_1_ compared to the “V”-shaped structure in the original P2_1_2_1_2 symmetry. The angle between the G helices of NpSRII in the “U”-shape and the “V”-shape is 11°. A single NpSRII monomer of the “U”-shape dimer can be superimposed with the corresponding monomer of the “V”-shape dimer by a rigid body rotation by an angle of 8.5° (see Fig. 4). These large differences between two quaternary structures might account for the altered HAMP domain-NpSRII binding interfaces. Unfortunately, the electron density in this region is too weak in both cases, presumably due to interactions of the proteins in the crystal lattice. However, molecular modeling revealed important details about interactions of the HAMP domain within the complex. We have prepared homology models of the NpHtrII HAMP domains and modeled their possible position relative to the transducer’s transmembrane part and to the receptor NpSRII starting from the known structures of the HAMP domains^6^,^24^,^25^ (PDB codes 1ASW and 3LNR). We found that only the “U”-shape structure allows a close interaction between NpSRII and the HAMP domain, allowing it to be compactly fitted into the “U”-shape topology (Fig. 5). On the other hand, the model based on the “V”-shaped topology does not show any contacts between the HAMP domain and NpSRII, which leads to weaker interactions between these proteins.

**Figure 6:**
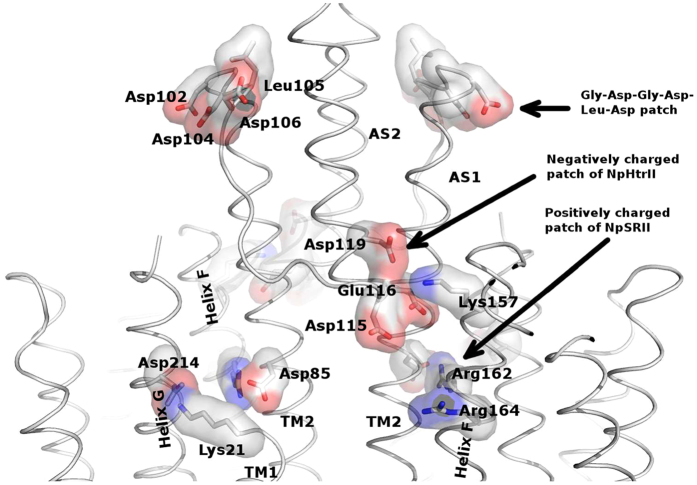
Electrostatic interactions between the receptor molecule and the HAMP domain. Interacting patches in the receptor and the transducer are shown and the G-D-G-D-L-D patch of the transducer proposed previously in literature as interacting with NpSRII. Positive charges are mapped in blue and negative are mapped in red. The expected salt bridges are: Asp214 (NpSRII) to Lys21 (NpHtrII), Lys157 (NpSRII) to Asp119 and Glu116 (NpHtrII).

The surface of NpSRII includes a unique group of charged and polar residues at the cytoplasmic ends of helix F (Lys157, Ser158, Arg162, Arg164, and Asn165) and G (Asp214) (Fig. 6). For Ser154 to Lys157 an interaction with the transducer has been proven by ssNMR experiments^26^. According to our model, this patch of amino acids could interact with Asp85, Ser91, Asp115, Glu116 and Asp119 on TM2 via electrostatic interactions. Furthermore, Lys21 of TM1 and Asp214 of the helix G of the receptor could form a salt bridge and might contribute to the overall stability of the complex conveyed by the HAMP domain-receptor interaction. The negatively charged cytoplasmic domain Gly101-Asp-Gly-Asp-Leu-Asp106 of TM2 of NpHtrII, which is conserved among various species^27^ and was suggested to interact with NpSRII previously^28^, apparently has no possible interactions with the receptor in our model (Fig. 6). We should also note that these results well explain the spectroscopic data that indicates that the EF loop interacts with the transducer^8^,^9^,^26^.

### Normal mode analysis

To further support our hypothesis that signal transduction may involve transition from the “U” to “V”-shaped complex structure, we have constructed an elastic network model (ENM)^29^ and performed a normal mode analysis (NMA) for the both “U” and “V”-shape structures as the starting models using the rotation translation block (RTB) approach^30^. The first two normal modes in both cases corresponded to the relative rotation of the subunits in the plane of the membrane. The third mode in corresponded to the “U”-to-“V” transition observed in the crystal structures. To see the effect of a lipid membrane, we repeated the NMA analysis for the NpSRII complex in the “U”-state inserted into a POPC bilayer consisting of 256 lipid molecules. We prepared the initial model using CHARMM-GUI^31^,^32^. Then, we equilibrated the bilayer for 1 ns using GROMACS v. 5.0^33^. The protein structure was fixed during the equilibration. In this case, we did not identify a single mode responsible for the “U”-to-“V” transition. However, using a linear combination of ten lowest modes, we could find a motion that reduced the overall RMSD of the transition from 2.9 Å to 1.0 Å.

**Figure 7:**
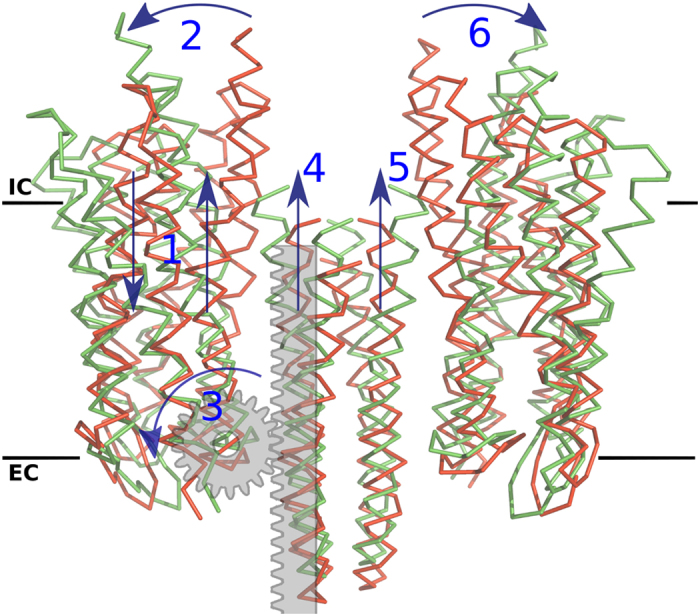
Normal mode analysis of the complex suggests a rack&pinion model for the signal transduction. Retinal isomerization leads to breaking of the receptor into two domains that move in the opposite directions perpendicular to the membrane (1); shift of its transmembrane helices relative to each other creates a hydrophobic mismatch that has to be compensated through a tilt rotation of NpSRII (2) around the intracellular point of contact between NpSRII and NpHtrII (3); this motion levers TM1 and TM2 of the conjugated NpHtrII for a piston-like shift towards the cytosol (4), which concomitantly drags the helices of the other protomer, reversing the mechanism in the symmetric counterpart of the receptor (5) and (6). Solid black lines illustrate the approximate membrane boundaries with “IC” designating the intracellular side and “EC” designate the extracellular side.

These finding show that the direction of the U-to-V transition coincides with one or several of temperature fluctuations in the complex, thereby requiring minimal energy to take place. We also clearly observe a piston-like shift of both transmembrane helices of the transducer with respect to NpSRII, which occurs concomitantly with the rotation of the receptors (Fig. 7). When starting from the “U”-shape structure, the complex transitions into a “V”-shape-like model, which closely resembles the crystal structure of the active state (overall RMSD 1.04 Å, also see Supplementary Movie 1).

## Discussion

Multiple experimental studies (calorimetric^10^, solution NMR^11^, EPR experiments^34^) have long predicted the tight binding interface between the transducer and the receptor. In the previously determined structure of the complex (space group P2_1_2_1_2) quaternary structure has a “V”-shape and there was no possibility of interaction between the NpHtrII’s HAMP1 domain and NpSRII. Here, we have presented the structure in the space group I2_1_2_1_2_1_, where the whole complex is more compact (quaternary structure has a “U”-shape) and the receptor can extensively interact with the transducer’s HAMP domain.

In contrast to the ground state, in the active state of the complex, the interaction between NpSRII and NpHtrII is considerably weakened (dissociation constants are 0.10 μM and 15 μM correspondingly)^35^,^36^. This effect can originate from a perturbation of the linker region. However, it cannot be caused by the transmembrane part of the proteins, since the interactions (especially, the hydrogen bonds interactions) are nearly the same in both states. Interestingly, the HAMP domain itself (NpHtrII^G83-Q149^) interacts with the receptor in the ground state, but not in the M state^11^. Since the HAMP domain is sterically quite constrained hampering conformational changes in the “U”-shape, it may be that a transition from “U” to “V”-shape is involved in the signal propagation.

We have recently demonstrated via molecular dynamics study that the HAMP domain adopts an asymmetric conformation in its resting state, in which the protomers are longitudinally shifted by 1.3 Å with respect to each other presumably causing a deviation of the cytoplasmic part of NpHtrII relative to its transmembrane part^6^. Thus, it may happen that the “U”- and “V”–shaped complexes just correspond to the symmetric and asymmetric states of the HAMP domains and, therefore, correspond to different functional states, namely the ground and the active state. We suggest that the “U”-shape corresponds to a symmetric state of the HAMP domain, where it is stabilized in the rotated conformation via the aforementioned electrostatic interactions with the receptor. In this state, the HAMP domain adopts a compact conformation which has been correlated with the active state of the complex^37^,^38^. This conformational state is made possible by a high flexibility of the linker, especially in the region of Gly83-Gly84 amino acid repeat. This hypothesis is supported by another well-established fact: the G83F mutation completely inhibits signal transduction^39^. Indeed, replacing a small glycine by a considerably larger phenylalanine would limit the ability of the HAMP domain to rotate relative to the membrane part of the transducer and thus forbids the ground state. In line with this reasoning, our data on the crystal structure of the NpSRII/NpHtrII_1–135_-G83F mutant demonstrate different crystal packing. Although the crystallization conditions were the same as for the wild type “U”-shaped complex in the P6_4_ space group, in this case the space group is P2_1_2_1_2 giving the “V”-shape structure similar to those observed before^12^.

Another evidence in favor of the functional significance of the “U”-shaped membrane protein NpSRII/NpHtrII complex was published recently^40^. The authors studied how photoexcitation of NpSRII affects the structures of the complex. They conducted two series of 90 nsec molecular dynamics simulations of the complex linked with a modeled HAMP domain in the lipid bilayer using the published “V”-shaped structures of the ground and the M intermediate states^12^,^13^ as starting models. The most significant finding was that the orientations of the two NpSRII molecules changed in going from the ground state to the M state: their ‘closed’ ground state can be correlated to the “U”-shaped topology introduced in this paper, whereas their more flexible M state might represent the “V”-shape.

Finally, a very similar transition has been observed in one of the histidine kinases, PhoQ, via disulfide crosslinking^41^. The PhoQ sensor belongs to the family of two-component signal transduction systems. Similarly to NpHtrII, it contains a HAMP domain and has an analogous TM domain^42^. TM domain, HAMP and periplasmic domains of PhoQ were investigated using disulfide-scanning mutagenesis. The obtained crosslinking data were best explained by the two-state modeling approach than the one-state modeling, where the signal is suggested to be transferred through scissor-like transition from one state to the other.

Thus, it seems that the mechanism suggested here does not appear to be unique for NpSRII/NpHtrII complex, but rather might be widespread among two-component signaling systems in general. It also does not contradict with the existing models of signal transduction through the HAMP domains. Molecular interactions between NpSRII and HAMP can transition the latter from the compact state to the splayed state as reported before^7^. Other models like partially disordered HAMP as in the dynamic bundle model^43^ or alternating dynamics of NPHtrII subdomains^38^ deal with the signal transfer from the membrane proximal region to the tip of the NPHtrII cytoplasmic and can be incorporated into our model of signal transfer from the membrane domain to the first HAMP domain (HAMP1).

## Concluding Remarks

Summarizing, the “U”- and “V”-shaped conformations of the NpSRII/NpHtrII complex may be crucial for the mechanism of signal propagation spanning the membrane domain and feeding into the HAMP domain. This work also represents one more evidence that different crystallization conditions and/or different crystal packing may force the protein to adopt different conformations corresponding to discrete functional states. Obviously, it is not a completely new observation; it was shown that the extracellular domain of metabotropic glutamate receptor can adopt an active-like conformation even in the absence of the ligand^14^. However, to the best of our knowledge, this is the first such demonstration for the case of membrane proteins where the different space groups could potentially trap different functional states of the transmembrane part of proteins and which might be important for signal transfer in arrays of receptors as it was proposed for chemoreceptors^44^.

## Methods

### Protein preparation

The NpSRII and NpHtrII proteins were produced in *E. coli*, purified, reconstituted into a functional complex, and resolubilized as described before^13^,^45^.

### Crystallization

We used a new *in meso*^46^ approach to obtain crystals. The complex in a crystallization buffer (150 mM NaCl, 25 mM Na/K phosphate, pH 5.1, n-octyl-b-D-glucopyranoside) was added to the lipid phase, formed on base of monooleoyl (Nu-Chek Prep). Best crystals were grown at 22 °C using 1 M Na/K phosphate, pH 5.6 and trehalose as a precipitant.

### Trapping of the M intermediate, data collection and refinement

The optimal procedure of the M state trapping was as described before^15^. X-ray diffraction data were collected at beamline ID14-1 of the European Synchrotron Radiation Facility (ESRF), Grenoble, France. Radiation dose was the same for the ground and M state and kept under reasonable limit to avoid influence of the radiation damage on the M state structure^47^. Data was integrated using MOSFILM^48^ and scaled with SCALA^49^ from the CCP4 program suite^50^. Molecular replacement was performed using MOLREP^51^ for a polyalanine model (from Protein Data Bank accession number 1H2S) and gave a unique solution. Structure refinement was done with Phenix^52^. The occupancy determination of the M state and the structure refinement was performed as before^13^.

### Modeling of the HAMP domain

In order to better understand the influence of the crystal packing on the NpHtrII structure, we modeled its possible continuation to the first HAMP domain (residues 83–136) in two space groups I2_1_2_1_2_1_ and P2_1_2_1_2, which is not observed in the crystal (Fig. 5). Based on the previous modeling studies for this region based on homology with the available HAMP domain structures^6^,^53^, we assumed that the linker between the helix TM2 and the HAMP domain adopts an alpha-helical conformation. The HAMP domain was modeled by homology (based on PDB code 2ASW). We optimized the models using a symmetry module from SAMSON modeling package^54^. Details of the modeling are provided in SI. We used PyMOL to calculate vacuum electrostatics potentials on molecular surfaces and to produce illustrations for this report^55^.

## Additional Information

**Accession codes:** Coordinates and structure factors have been deposited in the Protein Data Bank with the following accession numbers: 5JJE (NpSRII/HtrII-157 ground state), 5JJJ (NpSRII/HtrII-135 ground state), 5JJF (the M state of NpSRII/HtrII-157), 5JJN (NpSRII/HtrIIG83F-135).

**How to cite this article**: Ishchenko, A. *et al*. New Insights on Signal Propagation by Sensory Rhodopsin II/Transducer Complex. *Sci. Rep.* **7**, 41811; doi: 10.1038/srep41811 (2017).

**Publisher's note:** Springer Nature remains neutral with regard to jurisdictional claims in published maps and institutional affiliations.

## Supplementary Material

Supplementary Information

Supplementary Movie 1

## Figures and Tables

**Figure 1:**
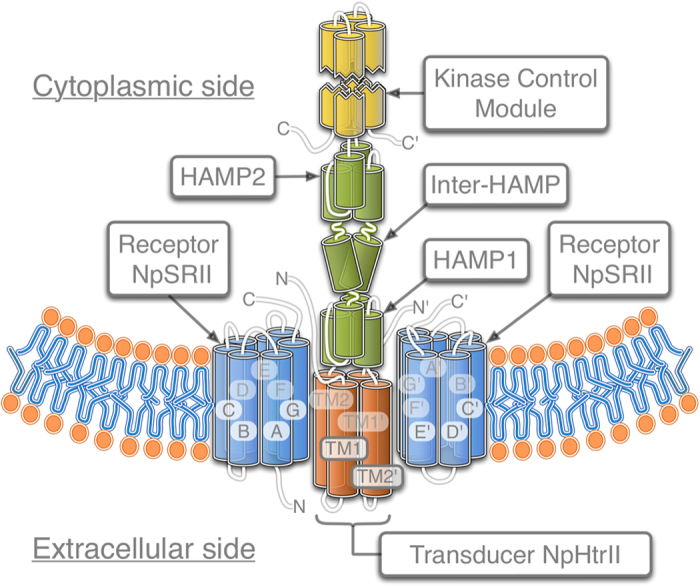
Domain architecture of the halobacterial NpSRII/NpHtrII complex. The dimer of two NpHtrII proteins is flanked by two NpSRII proteins. A-G, TM1 and TM2 are the transmembrane helices. Cytoplasmic part of NpHtrII consists of two HAMP domains (HAMP1 and HAMP2) connected by an α-helical linker (Inter-HAMP) and the kinase control module. Primes denote symmetry mates of the complex.
