# Supplementary material for: New Insights on Signal Propagation by Sensory Rhodopsin II/Transducer Complex
Source: Sci Rep. 2017 Feb 6;7:41811. doi: 10.1038/srep41811 (PMC5292967; doi:10.1038/srep41811)
Supplement: Supplementary Information [file srep41811-s1.pdf]

Supporting Information

New Insights on Signal Propagation by Sensory Rhodopsin II/Transducer Complex

A. Ishchenko[ab#], E. Round[acde], V. Borshchevskiy[cd], S. Grudinin[df], I. Gushchin[acde], J. P. Klare[gh], A. Remeeva[a], V.Polovinkin[a,c,d], P. Utrobin[a,c,d], T. Balandin[a], M. Engelhard[g], G. Büldt[a,e], and V. Gordeliy [abcde*]

Table 1. Crystallographic data collection and refinement statistics

	Ground State, HtrII truncated at resid. 157	M State, HtrII truncated at resid. 157	Ground State, HtrII truncated at resid. 135	Ground State, HtrII truncated at resid. 135 with point mutation G83F
**Data collection**				
Space group	$I2_12_12_1$	$I2_12_12_1$	$P6_4$	$P2_12_12_1$
Cell dimensions				
a, b, c (Å)	49.64, 113.66, 125.99	49.56, 113.78, 125.39	66.31, 66.31, 170.08	47.13, 109.10, 121.82
α, β, γ (°)	90, 90, 90	90, 90, 90	90,90,120	90,90,90
Resolution (Å)	28.0-1.9 (1.97-1.90*)	36.0-1.9 (1.97-1.90*)	34.0-2.5 (2.59-2.50*)	38.0-2.25 (2.32-2.25*)
Rmerge (%)	7.0 (39.3*)	5.1 (14.4*)	9.0 (39.6*)	12.3 (50.1*)
I / σI	13.4 (2.4*)	18.7 (3.8*)	11.16 (2.1*)	11.16 (2.0*)
Completeness (%)	99.4 (99.8*)	98.6 (95.6*)	77.6 (73.4*)	99.5 (99.8*)
Redundancy	3.8 (3.5*)	3.4 (3.3*)	5.2 (5.5*)	3.5 (3.30*)
**Refinement**				
Resolution (Å)	22-1.9	36-1.9	34-2.5	38.0-2.25
No. reflections	28430 (1423**)	28034 (1402**)	20997 (1080**)	30446 (3000**)
Rwork / Rfree (%)	19.66 / 21.83	20.15 / 23.24	19.32 / 23.37	18.84 / 22.96
No. atoms				
Protein	2090	4180 (2090***)	2034	4139
Ligand/ion	171	191 (171***)	110	250
Water	56	110 (54***)	26	49
**B-factors**				

Protein	22.70	24.30 (23.90***)	56.10	37.76
Ligand/ion	41.70	44.40 (48.50***)	69.70	54.64
Water	32.20	33.60 (32.90***)	59.70	46.36
R.m.s. deviations				
Bond lengths (Å)	0.011	0.010	0.012	0.010
Bond angles (°)	1.23	1.30	1.87	1.11

*Values in parentheses are for high-resolution shell.

** Number of reflection not used for refinement (free reflections)

*** Number of atoms whose position was refined (atoms of active-state model)

Rmerge=∑h∑i|I(h,i)-<I(h)>|/∑h∑i|I(h,i)|× 100 %, where I(h,i) is the intensity value of the ith measurement of h and <I(h)> is the corresponding mean value of h for all I measurements of h. The summation is over all measurements.

Rwork = ∑|Fo-Fc|/|∑Fo|× 100 %.

Rfree was calculated for 5% of observed reflections, omitted from the refinement and Rwork calculation and picked randomly within thin resolution shells.

Supplementary Video 1

The movie clip shows the transition in the complex structure according to the normal mode 3 in line with the suggested mechanism of signal propagation.

Materials and Methods

Protein preparation

The coding regions of the *N. pharaonis* SRII and C-terminal truncated transducer (1–157) genes were cloned into a pET27bmod expression vector in frame with a C-terminal His_7 tag. Proteins were expressed in *E. coli* strain BL21(DE3), and purified as described[1]. After imidazole removal by DEAE chromatography, SRII-His and HtrII-157-His were mixed in a 1:1 molar ratio, followed by the reconstitution into purple membrane (the bacteriorhodopsin containing membrane patches of *H. salinarum*) lipids (protein to lipid ratio 1:35) using BioBeads II for detergent removal. The reconstituted proteins were pelleted by centrifugation at 100,000g and solubilized with 2% n-octyl-b-D -glucopyranoside for 16 h at 4° C in the dark. The solubilized complex was used for crystallization after removal of insoluble material by centrifugation at 100,000g.

C-terminal truncated transducer (1–135) gene was cloned into pTXB1 expression vector. Protein was expressed and purified according to Impact kit (New England Biolabs) protocols.

Crystallization

We added the solubilized complex in crystallization buffer (150 mM NaCl, 25 mM Na/KPi, pH 5.1, 0.8% n-octyl-b-D -glucopyranoside) to the lipidic phase, formed from monovaccenin (Nu-Chek Prep). Precipitant was 1 M salt Na/K-Pi, pH 5.6. Crystals were grown at 22° C.

Trapping of the M intermediate

To obtain the highest yield in the occupancy of the M-state we always used the optimum wavelength and intensity of an argon ion laser. This procedure was well established during data collection of the M state of crystals of SRII alone and performed similarly for the SRII/HtrII complex crystals. For trapping the late M-state the crystal was left to warm up to about 20° C (by blocking the nitrogen cryostream), illuminated for 3 s with argon laser light at 488 nm (about 30 $mW \times mm^{-2}$), then cooled (by unblocking the cryostream) while the blue light was still on. One second after cooling started, the illumination was turned off. X-ray data were collected in the dark.

Data collection and refinement

X-ray diffraction data were collected at beamline ID14-1 of the European Synchrotron Radiation Facility (ESRF), Grenoble, France, using a Quantum ADSC Q4R CCD (charge-coupled device) detector. Data was integrated using MOSFILM[2] and scaled with SCALA[3] from the CCP4 program suite[4]. Molecular replacement was performed using MOLREP[5] for a polyalanine model (from Protein Data Bank accession number 1H2S) and gave a unique solution.

Starting from a polyalanine model of NpSRII/NpHtrII (Protein Data Bank accession code 1H2S) the molecular replacement solution was completed by the automated refinement procedure (ARP/wARP[6]) using ground state data. The data refinement was performed using Refmac5[7] and phenix.refine[8]. Residues 22–84 of the transducer were built.

Simulated annealing and individual B-factor refinement were used (CNS[9]). Several data sets from non-illuminated crystals gave almost identical models of the ground state with a mean coordinate variation of 0.1 Å. Difference density maps $\Delta\rho = (|F_{ill}| - |F_{Gobs}|)\exp(i\varphi_G)$ revealed major structural changes that occurred on illumination of the crystals, with $|F_{ill}|$ being the observed amplitudes from illuminated crystals, $|F_{Gobs}|$ the observed ground-state amplitudes and φ_G the phases calculated from the ground-state model. Refinements of intermediate states were performed by starting with a superposition of the fixed ground-state structure under refinement with occupancies varying in the range of α=0.2–0.8 by simulating annealing (using 3000 and 5000 K and a slow cooling protocol in CNS). Intermediate state occupancies were estimated from the best correlation as 50 ± 10% .These variations had no significant effect on the conformation for the intermediate state after the refinement. After occupancy had been fixed, models were further refined and manually rebuilt in program Coot until convergence of R factors was reached.

Modeling of the HAMP domain

In order to better understand the influence of the crystal packing on the NpHtrII structure, we modeled its possible continuation to the first HAMP domain (residues 83-136), which is not observed in the crystal. It is assumed that the linker between the transmembrane helix TM2 and the HAMP domain adopts an alpha-helical conformation (for more details see previous reports[10]). The HAMP domain was modeled by homology, based on the NMR structure of the Archeoglobus

fulgidis hypothetical protein Af1503 (PDB code 2ASW). The transmembrane part of the NpHtrII with the bound NpSRII receptor were taken from the crystal structure. The combined model was minimized using a symmetry module from SAMSON modeling package[11] in the corresponding symmetry space group, with alpha-helical constraints imposed on NpHtrII residues 78-87. All water molecules, fragments of lipids and cofactors found in the crystal structure were preserved during the structure optimization. Crystal packing was maintained with implicit crystallographic symmetry transforms applied according to the considered space group. Interactions within modeled molecules and its symmetrical replicas were computed using a smooth version of CHARM19 force field[12]. A distance cutoff value of 8 Å was used during the structure optimization. The optimization was performed using the steepest descent method in the dihedral subspace with random jumps from the local minima according to the Metropolis acceptance criterion. The local minimum was defined as a structure with all accelerations on its dihedral degrees of freedom smaller then 1x10-2 Å/fs2. Finally, the structure with the lowest energy was chosen as a final model.

The same procedure was employed for NpSRII-NpHtrII complex in another spacegroup, P21212 (PDB code 2F95). In this crystal structure, residues 27-79 (out of 4-159) of NpHtrII are resolved up to resolution of 2.2 Å. Because of severe steric clashes between the model of the HAMP domain in one crystal layer with residues 35-66 from the model of NpHtrII on the other crystal layer, in order to preserve the folding of the HAMP domain we had to remove NpHtrII molecules on every second crystal layer.

We used PyMOL software to calculate vacuum electrostatics potentials on molecular surfaces, as well as to produce illustrations for this study[13].

References

1. Hohenfeld, I. P., Wegener, A. A. & Engelhard, M. Purification of histidine tagged bacteriorhodopsin, pharaonis halorhodopsin and pharaonis sensory rhodopsin II functionally expressed in Escherichia coli. *FEBS Lett.* **442,** 198–202 (1999).
2. Leslie, A. G. W. The integration of macromolecular diffraction data. *Acta Crystallogr. D. Biol. Crystallogr.* **62,** 48–57 (2006).
3. Evans, P. Scaling and assessment of data quality. *Acta Crystallogr. D. Biol. Crystallogr.* **62,** 72–82 (2006).
4. Collaborative Computational Project, N. 4. The CCP4 suite: programs for protein crystallography. *Acta Crystallogr. D. Biol. Crystallogr.* **50,** 760–3 (1994).
5. Vagin, A. & Teplyakov, A. MOLREP : an Automated Program for Molecular Replacement. *J. Appl. Crystallogr.* **30,** 1022–1025 (1997).
6. Langer, G., Cohen, S. X., Lamzin, V. S. & Perrakis, A. Automated macromolecular model building for X-ray crystallography using ARP/wARP version 7. *Nat. Protoc.* **3,** 1171–9 (2008).
7. Murshudov, G. N., Vagin, A. A. & Dodson, E. J. Refinement of macromolecular structures by the maximum-likelihood method. *Acta Crystallogr. D. Biol. Crystallogr.* **53,** 240–55 (1997).
8. Adams, P. D. *et al.* PHENIX: a comprehensive Python-based system for macromolecular structure solution. *Acta Crystallogr. D. Biol. Crystallogr.* **66,** 213–21 (2010).
9. Brünger, A. T. *et al.* Crystallography & NMR system: A new software suite for macromolecular structure determination. *Acta Crystallogr. D. Biol. Crystallogr.* **54,** 905–21 (1998).
10. Gushchin, I. Y., Gordeliy, V. I. & Grudinin, S. Role of the HAMP Domain Region of Sensory Rhodopsin Transducers in Signal Transduction. *Biochemistry* **50,** 574–580 (2010).
11. Grudinin, S. & Redon, S. Practical modeling of molecular systems with symmetries. *J. Comput. Chem.* **31,** 1799–1814 (2010).

12. Brooks, B. R. *et al.* CHARMM: A program for macromolecular energy, minimization, and dynamics calculations. *J. Comput. Chem.* **4,** 187–217 (1983).

13. DeLano, W. L. The PyMOL Molecular Graphics System, Version 1.2r3pre, Schrödinger, LLC.
